# Butyrate alleviates food allergy by improving intestinal barrier integrity through suppressing oxidative stress‐mediated Notch signaling

**DOI:** 10.1002/imt2.70024

**Published:** 2025-04-03

**Authors:** Jialu Shi, Wenjun Mao, Yuqing Song, Yuxin Wang, Lili Zhang, Yan Xu, Huiwen Gu, Siyu Yao, Yuanhang Yao, Zhifeng Liu, Vijaya Raghavan, Jin Wang

**Affiliations:** ^1^ Key Laboratory of Environmental Medicine and Engineering, Ministry of Education, Department of Nutrition and Food Hygiene, School of Public Health Southeast University Nanjing China; ^2^ Department of Thoracic Surgery, The Affiliated Wuxi People's Hospital of Nanjing Medical University, Wuxi People's Hospital, Wuxi Medical Center Nanjing Medical University Wuxi China; ^3^ Department of gastroenterology Children's Hospital of Nanjing Medical University Nanjing China; ^4^ Department of Bioresource Engineering, Faculty of Agricultural and Environmental Sciences McGill University, Sainte‐Anne‐de‐Bellevue Montreal Quebec Canada

**Keywords:** food allergy, gut microbiota, intestinal epithelial cells, Notch signaling, oxidative stress, short‐chain fatty acids

## Abstract

Food allergy (FA) has received increased attention in recent years. Multiple studies have highlighted the crucial role of short‐chain fatty acids (SCFAs) in the development of IgE‐mediated FA. Here, a case‐control approach was employed to analyze SCFAs profiles in children with FA, while an ovalbumin (OVA)‐sensitized mouse model was utilized to explore the underlying mechanism by which SCFAs mitigate FA. Children with food‐sensitized tolerance (FST) (*n* = 20) or FA (*n* = 20), and healthy controls (HC) (*n* = 20) were recruited to analyze SCFAs profiles. The HC group exhibited higher SCFAs levels in fecal samples than the FST, FA, and FST + FA groups. Data from an OVA‐sensitized mouse model showed that butyrate exhibited a more significant effect on reducing allergic reactions compared to other SCFAs. Compared to the negative control group, OVA‐induced oxidative stress (OS) triggered excessive Notch signaling activation, which subsequently impaired both tight junctions integrity and mucosal barrier function in murine intestinal epithelial cells (IECs). Gut dysbiosis induced mucus layer erosion, thereby elevating IECs exposure to food antigens and OS, which potentiated Notch signaling activation. However, butyrate counteracted this loop by restoring microbiota structure and suppressing reactive oxygen species (ROS)/Notch cascades. Strikingly, low‐dose butyrate (0.25–1 mM) protected rat small intestine crypt epithelial cells (IEC‐6) by inhibiting ROS, whereas high‐dose (2–5 mM) exacerbated oxidative injury and triggered activation of Notch signaling. Our study revealed the potential molecular mechanisms through which butyrate alleviates food allergy, providing a potential therapeutic strategy for its management.

## Highlights

- Children with food‐sensitized tolerance and food allergy exhibited significantly lower levels of fecal short‐chain fatty acids (SCFAs) compared with healthy controls.
- Ovalbumin‐sensitized mice revealed butyrate alleviated food allergy most effectively among SCFAs.
- Butyrate reduced reactive oxygen species production, thereby suppressing Notch signaling in intestinal epithelial cells and enhancing gut barrier function.

## Graphical abstract

Compared to healthy controls, children with food‐sensitized tolerance and food allergy showed reduced fecal short‐chain fatty acids (SCFAs), particularly butyrate. In an ovalbumin‐sensitized mouse model, butyrate outperformed other SCFAs in alleviating allergic responses. Butyrate suppressed reactive oxygen species production in intestinal epithelial cells, downregulated the Notch ligand Jagged1 expression, and reduced Notch intracellular domain nuclear translocation, thereby inhibiting the Notch downstream gene *Hes1*. Consequently, enhanced tight junctions protein expression improved barrier integrity, limiting food allergen permeation and subsequent Th2 immune responses. This attenuated mast cell degranulation (e.g., histamine and mouse mast cell protease 1 (mMCP‐1)) and alleviated allergy symptoms. The study highlighted the therapeutic potential of butyrate‐enhancing strategies‐including dietary fibers, prebiotics, and probiotics‐as modalities for allergy prevention or treatment.

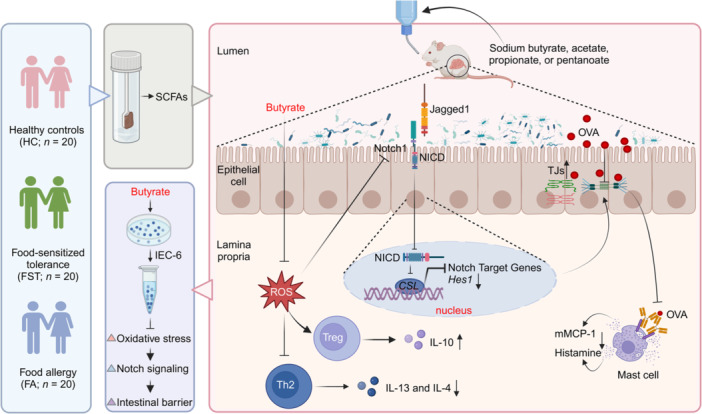

## INTRODUCTION

The global incidence of food allergy (FA) has risen strikingly in recent decades. The study indicated that approximately 8% of children and 5% of adults worldwide suffer from FA [1]. FA arises from a breakdown in oral tolerance—an immune regulatory mechanism mediated by gut‐associated lymphoid tissues that normally prevents hypersensitivity to dietary antigens [2]. Although any food protein can be allergenic, the most common triggers include milk, eggs, wheat, soy, sesame, peanut, crab, shrimp, and nuts [3]. FA manifests across multiple organ systems, ranging from gastrointestinal disturbances, cutaneous reactions, and respiratory symptoms to life‐threatening anaphylactic shock [4]. To date, no curative therapies exist, making strict allergen avoidance the cornerstone of management for individuals with FA. Tolerance to food antigens begins with their absorption in the small intestine, where intestinal epithelial cells (IECs) serve a pivotal role by forming barrier. Their tight junctions (TJs) establish a physicochemical defense, while secreted antimicrobial peptides and mucus create an immunological shield that compartmentalizes commensal microbiota and limits pathogenic invasion [5]. The reciprocal interactions between gut microbiota and IECs are essential for maintaining immune homeostasis at the mucosal interface, orchestrating a balance between pro‐inflammatory and tolerogenic responses [6]. This symbiosis not only modulates intestinal immune equilibrium through microbial metabolite production, such as short‐chain fatty acids (SCFAs), and IECs‐derived cytokine signaling but also critically determines whether immune responses skew toward tolerance or hypersensitivity to dietary antigens. The maintenance of the intestinal barrier integrity, including mucosal and intercellular TJs, has been associated with a number of disease states [7]. Barrier disruption enables paracellular translocation of luminal antigens (e.g., microbial components, dietary allergens) into the lamina propria, where they activate innate immune sensors to trigger inflammatory cascades [8]. Beyond its canonical role in development, the Notch signaling pathway critically modulates intestinal epithelial homeostasis, orchestrating cell fate decisions and barrier repair mechanisms [9, 10]. Notably, Notch inhibition attenuates gut hyperpermeability via enhanced TJs assembly [11]. However, the Notch signaling in FA‐induced barrier remodeling remains to be fully evaluated.

Recent studies suggested that an imbalance in the gut microbiota may contribute to the development of FA. Targeted modulation of the gut microbiota may offer novel preventive strategies for FA. The gut microbiota produces a wide range of complementary enzymes with various specificities, which collectively catalyze dietary polysaccharide depolymerization and fermentation into SCFAs accessible for host absorption [12]. SCFAs (primarily acetate, propionate, butyrate, and valerate) exert protective effects against allergic diseases [13]. SCFAs regulate immune homeostasis by interacting with dendritic cells and T lymphocytes, suppressing pro‐inflammatory cytokine secretion, enhancing TJs protein expression, and reducing paracellular permeability [14, 15]. Despite their therapeutic potential, methodological heterogeneity in SCFAs quantification (e.g., extraction protocols, analytical techniques) and interindividual variability (e.g., diet, host genetics) challenge the interpretation of fecal SCFAs levels. The precise mechanisms by which SCFAs regulate Notch signaling in IECs to maintain barrier integrity during allergic sensitization remain poorly defined.

The aim of this study was to delineate the role of gut microbiota‐derived SCFAs in FA pathogenesis and intervention. We first performed a comparative metabolomic analysis of fecal SCFAs profiles (propionic acid, acetic acid, pentanoic acid, and butyric acid) in the feces of children with FA and food‐sensitized tolerance (FST) to those of healthy controls (HC). Subsequently, preclinical models of ovalbumin (OVA)‐induced FA were employed to quantify efficacy of individual SCFAs supplementation. To unravel the tripartite interplay delineated microbial‐epithelial‐immune crosstalk, we adopted a multi‐omics approach combining 16S rRNA sequencing, targeted metabolomics, and transcriptomic profiling of IECs. In addition, Notch signaling activity in rat small intestine crypt epithelial cells (IEC‐6) was modulated by SCFAs to determine the causal relationship with SCFAs‐mediated barrier restoration and immune tolerance. Our systematic strategy provided new insights into FA prevention.

## RESULTS

### Participant characteristics and SCFAs assessments in fecal samples

**Figure 1:**
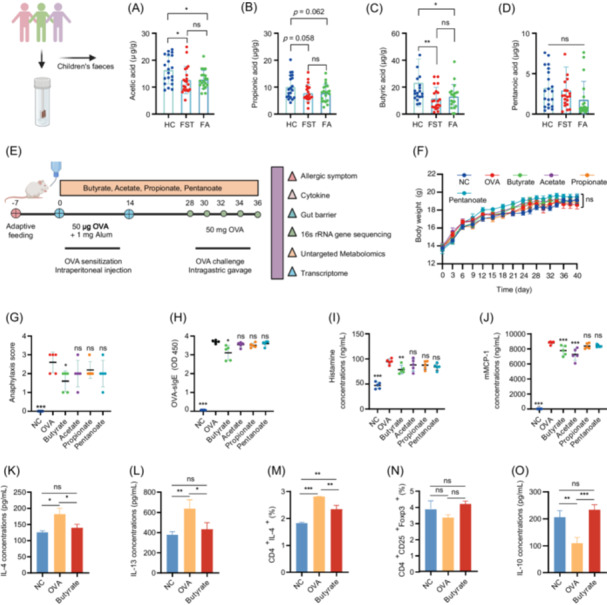
Short‐chain fatty acids (SCFAs) concentrations of children's fecal samples and the effect of SCFAs on food allergy in ovalbumin (OVA)‐sensitized mice. The concentrations of acetic acid (A), propionic acid (B), butyric acid (C), and pentanoic acid (D) in HC, FST, and FA groups, *n* = 20. (E) The experimental design of the food allergy mouse model. Four‐week‐old female BABL/c mice were used in the FA animal model. At the start of sensitization, sodium butyrate, acetate, propionate, or pentanoate was administered in drinking water at 200, 100, 100, and 100 mM, respectively, which continued throughout the study. On Days 0 and 14, mice were sensitized via intraperitoneal injection of 50 μg OVA with 1 mg aluminum potassium sulfate as adjuvant. The control mice receive aluminum potassium sulfate only. On Days 28, 30, 32, 34, and 36, mice were orally challenged with 50 mg of OVA. (F) Body weight was measured throughout the experiment. (G) Anaphylaxis scores were assessed for 1 h after the last challenge, and serum samples were collected, *n* = 5. The levels of OVA‐specific IgE (OVA‐sIgE) (H), histamine (I), and mouse mast cell protease 1 (mMCP‐1) (J) in serum, *n* = 5. Interleukin‐4 (IL‐4) (K) and IL‐13 (L) cytokine secretion of splenocytes were assessed in the presence of OVA, *n* = 5. IL‐4‐producing CD4^+^ T cells (M) and CD4^+^CD25^+^Foxp3^+^ Treg cells (N) numbers of splenocytes were measured by flow cytometry after stimulation with phorbol‐12‐myristate‐13‐acetate (PMA)/ionomycin in the presence of brefeldin A, *n* = 3. (O) IL‐10 cytokine secretion of splenocytes was assessed in the presence of OVA, *n* = 5. Acetate, acetate group; butyrate, butyrate group; FA, food allergy group; FST, food‐sensitized tolerance group; HC, healthy controls group; NC, negative control group; OVA, OVA group; propionate, propionate group; pentanoate, pentanoate group. *p < 0.05; **p < 0.01; ***p < 0.001.

The concentrations of SCFAs in the stool samples from individuals with FA and FST were evaluated and compared to the levels in age‐matched nonallergic HC. Table S1 presented the demographic characteristics of the study population. The concentrations of acetic acid, propionic acid, butyric acid, and pentanoic acid were lower in the FA and FST groups, compared to the HC group (Figure 1A–D). Compared with the HC group, acetic acid and butyric acid were significantly reduced in FST and FA groups (Figure 1A,C). Notably, no significant differences in SCFAs concentrations were observed between the FST and FA groups (Figure 1A–D). The FST + FA (food allergen sIgE ≥ 0.35 KU_A_/L) group showed lower levels of SCFAs than the HC group (Figure S1A–D). Additionally, acetic acid and butyric acid were significantly downregulated in the FST + FA group compared to the HC group (Figure S1A,C). Propionic acid was partly reduced in FST, FA, and FAT + FA groups compared to that in the HC group (Figure 1B, Figure S1B). The above results indicated that SCFAs may have a protective role in FA.

### Butyrate protected against food allergy in OVA‐sensitized mice

The consumption of SCFAs is thought to promote human health and provide protection against FA. Based on this, we hypothesized that different SCFAs may have varying effects in reducing food allergic responses. As demonstrated in Figure 1F, OVA sensitization did not change the body weight of mice in all groups. However, mice in the OVA group exhibited more severe anaphylactic symptoms (Figure 1G) and showed significantly increased levels of OVA‐specific IgE (OVA‐sIgE) in serum (Figure 1H). To assess the impact of SCFAs supplementation on mast cell degranulation, which directly correlates with histamine and mouse mast cell protease 1 (mMCP‐1) release, we quantified serum concentrations of these mediators following oral challenge. Serum levels of histamine and mMCP‐1 were significantly elevated in OVA‐sensitized mice compared to the negative control (NC) group (Figure 1I,J). However, the introduction of butyrate reduced the clinical score, OVA‐sIgE levels, histamine levels, and mMCP‐1 levels relative to the OVA group (Figure 1G–J). Furthermore, while acetate, propionate, and pentanoate all exhibited a tendency to decrease allergy parameters, only acetate significantly decreased mMCP‐1 levels (Figure 1G–J). These results indicated that butyrate had a more potent effect in alleviating FA symptoms.

Next, we assessed butyrate's immunomodulatory effects by analyzing splenocyte cytokine profiles. Compared to NC, OVA‐sensitized mice exhibited a Th2‐skewed response, identified by an increase in IL‐4 and IL‐13 production (Figure 1K,L). However, butyrate supplementation inhibited the secretion of IL‐4 and IL‐13 (Figure 1K,L). Similar results were observed for IL‐4 expression in CD4⁺ T cells isolated from splenocytes (Figure 1M, Figure S2A). Moreover, OVA sensitization reduced splenic CD4^+^CD25^+^Foxp3^+^ Tregs frequency, which was rescued by butyrate treatment (Figure 1N, Figure S2B). This Tregs recovery correlated with elevated IL‐10 levels in butyrate‐treated mice (Figure 1O). These data confirmed the effect of butyrate in immunomodulation.

### Butyrate maintained intestinal mucosal physical barrier function

**Figure 2:**
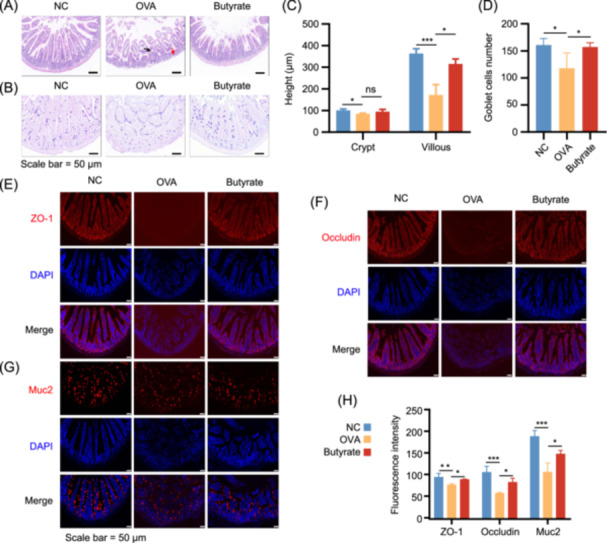
Butyrate maintained gut tight junctions (TJs) integrity and mucosal barrier function. Representative images of hematoxylin‐eosin (H&E) (A) and alcian blue‐periodic acid schiff (AB‐PAS) (B) staining of jejunum tissue. Crypt and villous height (C) and goblet cells (GCs) number (D) were measured in jejunum tissues. Representative immunofluorescence images of ZO‐1 (E), Occludin (F), and Muc2 (G) in jejunum tissue. (H) The immunofluorescence quantitative analysis of ZO‐1, Occludin, and Muc2 in jejunum tissue. Red arrow, crypt loss; Black arrow, villus collapse; NC, negative control group; OVA, OVA group; Butyrate, butyrate group, *n* = 3. *p < 0.05; **p < 0.01; ***p < 0.001.

We evaluated the effect of butyrate on intestinal barrier integrity and the mucus layer in the OVA‐induced FA (Figure 2). Hematoxylin‐eosin (H&E) staining results showed that the OVA group had significant damage to the intestinal mucosal structures, including crypt loss (red arrows) and villus collapse (black arrows) in the jejunum tissue. This damage was ameliorated after butyrate treatment (Figure 2A). Moreover, alcian blue‐periodic acid schiff (AB‐PAS) staining revealed damage to the intestinal mucosa, with villous epithelial cells in the jejunum tissue of OVA‐sensitized mice being disorganized and exfoliated (Figure 2B). And crypt and villous height, and goblet cells (GCs) number in the OVA mice were also substantially lower compared to NC mice (Figure 2C,D). However, villous height was increased with butyrate treatment, but no effect on crypt height was observed in the Butyrate group (Figure 2C,D). Immunohistochemical staining revealed diminished TJs protein expression in jejunum tissue. Notably, butyrate strikingly significantly increased the expression of ZO‐1 and Occludin in contrast with the OVA group (Figure 2E,F,H). In addition, Muc2 expression in jejunum tissue was reduced in the OVA group compared to NC controls but markedly elevated after butyrate treatment, consistent with GCs number results (Figure 2G,H). Collectively, butyrate improves intestinal barrier integrity by restoring the levels of TJs and Muc2 proteins.

### Butyrate treatment modulated gut microbiota in OVA‐sensitized mice

**Figure 3:**
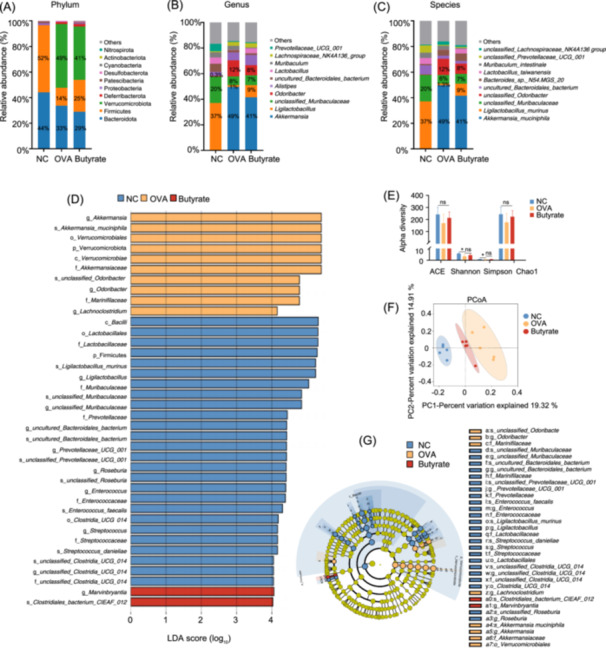
Butyrate treatment modulated gut microbiota in OVA‐induced mice. Gut microbial composition at the phylum (A), genus (B), and species (C) level in the feces sample. (D) The Linear discriminant analysis (LDA) composition of the microbiota (LDA score (log_10_) > 4). (E) Effects on the alpha diversity index. (F) Bray–curtis principal coordinate analysis (PCoA) of the beta diversity of OTUs of the gut microflora. (G) LDA effect size (LEfSe) of microbial comparison (LDA score (log_10_) > 4). Butyrate, butyrate group; NC, negative control group; OVA, OVA group, *n* = 5. *p < 0.05.

The fecal microbiota composition was analyzed to examine the effect of butyrate on bacterial composition (Figure 3). Compared to the NC group, OVA significantly decreased the alpha diversity (Shannon and Simpson index) of the fecal microbiota. Butyrate supplementation increased alpha diversity, but unfortunately, this change was not significant (Figure 3E). As shown in Figure 3F, β‐diversity indicated gut microbiota structure in the intestinal microbial composition among the groups by principal co‐ordinates analysis. The differentiation of the groups into separate clusters, driven by PC1 (19.32%) and PC2 (14.91%) factors, substantiates the observed disparities in the mice's gut microbiota.

Additionally, OVA administration resulted in a reduction in the Firmicutes/Bacteroidota (F/B) ratio, alongside decreased abundances of the species *Ligilactobacillus murinus* (*L. murinus*) and the unclassified *Muribaculaceae*. Conversely, the relative enrichment of the phylum Verrucomicrobiota, along with the species *Akkermansia muciniphila* (*A. muciniphila*) and the genus *Odoribacter*, was observed in comparison to the NC group (Figure 3A–C). However, butyrate intervention reversed this change to some extent. LDA and LEfSe analysis were performed to compare differences in the fecal bacterial community composition and determine biomarkers with statistically significant variations between the different groups. For OVA exposure, the species *A. muciniphila*, genus *Odoribacter*, and genus *Lachnoclostridium* were recognized as biomarkers in the OVA group (Figure 3D,G). After butyrate supplementation, the genus *Marvinbryantia* and the species *Clostridiales_bacterium_CIEAF_ 012* were identified as biomarkers. These data suggested that butyrate treatment could reshape the gut microbiome structure in OVA‐sensitized mice.

### The effect of butyrate treatment on the gut metabolites in OVA‐induced mice

**Figure 4:**
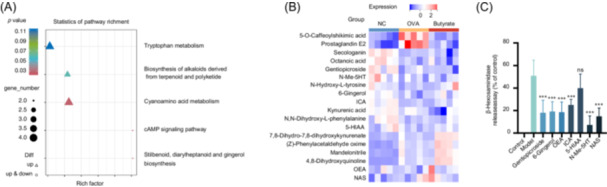
The effect of butyrate treatment on the gut metabolites in OVA‐induced mice and antiallergic properties of key gut metabolites. (A) Kyoto Encyclopedia of Genes and Genomes (KEGG) enrichment analysis from OVA vs Butyrate group was shown. (B) Relative levels of 18 differentially expressed metabolites (DEMs) in the major KEGG‐enriched pathways shown in the heatmap. (C) The inhibitory effects on β‐hexosaminidase release of key metabolites. Twenty‐hours after seeding, the cells were incubated with egg‐allergic patient's serum (1:25 dilution) for 24 h. The sensitized RBL‐2H3 cells were then treated with 50 μM indicated metabolites for 1 h and challenged with 30 μg/mL OVA for 1 h. Subsequently, the supernatant was collected to assess β‐hexosaminidase release, *n* = 3. ***p < 0.001.

Given the changes in gut microbiota, we examined whether butyrate had shaped the gut metabolome by using UPLC‐MS. Significant differences in fecal metabolites among the NC, OVA, and Butyrate groups were revealed by principal component analysis (PCA), indicating distinct metabolic profiles across these groups (Figure S3A). OPLS‐DA analysis demonstrated both model suitability (*Q* > 0.5 in all comparison groups) and distinct separations across groups (Figure S3B). These results suggest that consistent with gut microbes, intervention with butyrate alters the composition of intestinal metabolites. Fold change (FC) > 1, *p* ≤ 0.05, and variable projection importance > 1 as the threshold, 1452 differentially expressed metabolites (DEMs) were identified in the NC vs OVA groups, and 362 DEMs were identified in the OVA vs Butyrate groups (Figure S3C,D). Using the Kyoto Encyclopedia of Genes and Genomes (KEGG) database as a framework, we performed a KEGG pathway enrichment analysis on DEMs. Results showed that diarylheptanoid, stilbenoid, and gingerol biosynthesis [ko00945], cAMP signaling pathway [ko04024], cyanoamino acid metabolism [ko00460], biosynthesis of alkaloids derived from terpenoid and polyketide [ko01066], and tryptophan metabolism [ko00380] were the main significantly enriched KEGG pathways between OVA and Butyrate (Figure 4A). Figure 4B demonstrated DEMs in KEGG pathways; butyrate supplementation upregulated representative anti‐inflammatory and antioxidant metabolites (gentiopicroside, 6‐Gingerol, oleoylethanolamide (OEA)) and tryptophan metabolism‐related metabolites (indole‐3‐carboxaldehyde (ICA), 5‐Hydroxyindoleacetic acid (5‐HIAA), N‐Methylserotonin (N‐Me‐5HT), N‐Acetylserotonin (NAS)). The above data suggested that the intervention of butyrate upregulates the anti‐inflammatory, antioxidant, and tryptophan metabolites in OVA‐sensitized mice.

To determine whether these key metabolites possess antiallergic properties, we subsequently measured their inhibitory influences on β‐hexosaminidase release in the OVA‐induced rat basophil leukemia cells (RBL‐2H3) cell degranulation model. The cytotoxicity was assessed by cell counting kit‐8 assay (CCK8) (Figure S4). None of the key metabolites in the range of 0–50 μM induced significant cytotoxicity after 24 h of treatment compared to the control group. However, certain key metabolites at higher concentrations (100–300 μM) resulted in significant cytotoxicity (Figure S4A). Therefore, all key metabolites with a concentration of 50 μM were selected in subsequent experiments. After 24 h of treatment, OVA (20, 30, and 40 μg/mL) and egg‐allergic patient serum (1:15–1:25 dilution) did not induce significant cytotoxicity compared to the control group (Figure S4B,C). When 1:25 diluted serum sensitization was combined with 30 µg/mL OVA stimulation, RBL‐2H3 cells achieved the maximum release rate of β‐hexosaminidase and did not show significant cytotoxicity (Figure S4D,E). Therefore, this dose will subsequently be used for degranulation release modeling in RBL‐2H3 cells. The findings revealed that 50 μM gentiopicroside, 6‐Gingerol, OEA, ICA, N‐Me‐5HT, and NAS significantly reduced in the release of β‐hexosaminidase, with 5‐HIAA also evincing a propensity to diminish β‐hexosaminidase release (Figure 4C). These data indicated that butyrate supplementation could increase gut metabolites with anti‐allergic properties in OVA‐sensitized mice.

### Butyrate improved intestinal barrier integrity via inhibiting oxidative stress (OS)‐mediated Notch signaling

**Figure 5:**
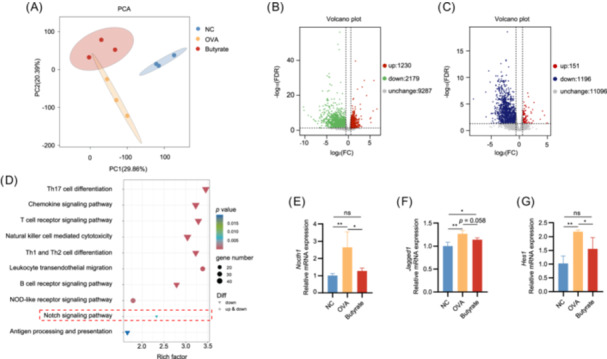
Butyrate significantly altered intestinal epithelial cells (IECs) gene expression in ovalbumin (OVA)‐induced mice. (A) Principal component analysis (PCA) showed that all IECs metabolites were significantly distinct in NC, OVA, and Butyrate groups. Significant differentially expressed genes (DEGs) between two groups: NC vs. OVA (B) and OVA vs Butyrate (C) (false discovery rate (FDR) < 0.05 & fold change (FC) ≥ 1.5) in the volcano plot. (D) KEGG enrichment analysis from the OVA to Butyrate group was shown. (E–G) Key genes in the Notch pathway in IECs were validated by quantitative real‐time PCR (qRT‐PCR). The mRNA expression of *Notch 1* (E), *Jagged1* (F), and *Hes1* (G) in IECs. NC, negative control group; OVA, OVA group; Butyrate, butyrate group, *n* = 5. *p < 0.05; **p < 0.01.

To investigate the molecular mechanisms behind the alleviating effect of butyrate on OVA‐induced FA, the gene expression of IECs was analyzed using RNA sequencing. PCA revealed significant differences in the gene expression profiles of IECs across all groups, suggesting that gene expression patterns varied notably among the groups (Figure 5A). A total of 3409 and 1347 differentially expressed genes (DEGs) were obtained by setting false discovery rate (FDR) ≤ 0.05 and FC ≥ 1.5 as a threshold in NC vs OVA groups and OVA vs Butyrate groups (Figure 5B,C). KEGG pathway enrichment analysis showed that the DEGs were enriched in several immunology‐related pathways (Figure 5D), such as Th17 cell differentiation [ko04659], Chemokine signaling pathway [ko04062], T cell receptor signaling pathway [ko04660], Th1 and Th2 cell differentiation [ko04658], Natural killer cell mediated cytotoxicity [ko04650], Leukocyte transendothelial migration [ko04670], B cell receptor signaling pathway [ko04662], Antigen processing and presentation [ko04612], NOD‐like receptor signaling pathway [ko04621], and Notch signaling pathway [ko04330]. The results demonstrated that the intervention of butyrate change the transcription level of genes in IECs.

KEGG pathway enrichment analysis revealed that butyrate suppressed the activation of the Notch signaling pathway in comparison to the OVA group (Figure 5D). In the present study, validation through qPCR revealed upregulation of the mRNA expression of Notch signaling pathway‐associated genes *Notch1*, *Jagged1*, and *Hes1* in IECs of OVA group mice. As expected, the levels of these genes were markedly diminished in mice subjected to butyrate treatment (Figure 5E–G).

**Figure 6:**
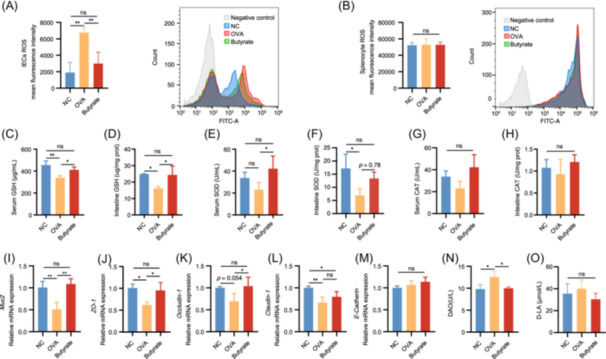
Butyrate improved intestinal barrier integrity via inhibiting oxidative stress (OS)‐mediated Notch signaling. Reactive oxygen species (ROS) mean fluorescence intensity IECs (A) and splenocyte (B) were measured by Flow Cytometry. The expressions of antioxidant‐related proteins were quantified. The levels of glutathione (GSH) (serum (C); small intestine (D)), superoxide dismutase (SOD) (serum (E); small intestine (F)), and catalase (CAT) (serum (G); small intestine (H)). (I–M) The mRNA expressions of TJs protein and mucin were quantified in IECs. The mRNA expressions *Muc2* (I), *ZO‐1* (J), *Occludin‐1* (K), *Claudin‐1* (L), and *E‐Cadherin* (M) in IECs. The levels of diamine oxidase (DAO) (N) and d‐lactic acid (d‐LA) (O) in serum. IECs, intestinal epithelial cells. Butyrate, butyrate group; NC, negative control group; OVA, OVA group, *n* = 3. *p < 0.05; **p < 0.01.

Considering that high concentrations of exogenous ROS are known to upregulate Notch1 [13], we further explored the effect of OVA and butyrate on OS. OVA elicited ROS overproduction in IECs. In contrast, butyrate treatment exerted a marked inhibition on ROS generation (Figure 6A). However, the same changes were not observed in the levels of ROS in splenic cells (Figure 6B). Compared to the NC group, OVA exposure was found to trigger OS, leading to decreased levels of antioxidant molecule (glutathione (GSH)), and enzymes (superoxide dismutase (SOD) and catalase (CAT)) in both serum and small intestine homogenates (Figure 6C–H). In contrast with the OVA group, a significant upregulation of GSH and SOD was observed in serum after butyrate treatment (Figure 6C,E). Moreover, a significant increase was observed in GSH levels in the small intestine homogenate and a tendency toward increasing SOD levels (Figure 6D,F). However, there was no change in the concentration of CAT in either the serum or the small intestine homogenate (Figure 6G,H). This suggested that activation of Notch signaling could be associated with OVA‐induced OS.

The Notch signaling pathway acts a significant role in determining IECs fate and modulating gene transcription to coordinate GCs differentiation and MUC biosynthesis. Therefore, we investigated the regulatory role of OS‐associated Notch signaling pathway in intestinal barrier impairment. This study found that OVA exposure led to mucus barrier injury in IECs, resulting in reduced *Muc2* expression, whereas *Muc2* was upregulated in butyrate‐treated mice (Figure 6I). Compared to the controls, similar results were obtained in the expression of TJs‐associated protein, such as *ZO‐1*, *Occludin‐1*, and *Claudin‐1*, and *E‐Cadherin* of IECs, were inhibited in OVA‐induced mice, except for *E‐Cadherin* levels, which were unaltered. However, it was attenuated by butyrate treatment (Figure 6J–M). It was observed that butyrate intervention significantly decreased the OVA‐induced elevation of diamine oxidase (DAO) levels, while the d‐lactic acid (d‐LA) levels did not show significant changes in any group (Figure 6N,O). This suggested a mechanism by which butyrate improves intestinal barrier integrity via inhibiting OS‐mediated Notch signaling, in turn, reducing the absorption of food allergen and immune responses.

### Dose‐dependent effects of butyrate on OVA‐induced OS and Notch signaling

To establish the role of OS in OVA‐induced intestinal barrier dysfunction, IEC‐6 cells were treated with OVA for 24 h (Figure S5A). OVA aggravated OS in IEC‐6, as demonstrated by the increase in ROS production (Figure S5B). The levels of GPH, SOD, and CAT, which are classical indicators of OS, were significantly decreased following OVA stimulation (Figure S5C–E). Figure S5F,G revealed that the mRNA expression of Notch signaling pathway‐associated genes *Jagged1* and *Hes1* were significantly increased after OVA exposure. Notably, OVA induced IEC‐6 barrier damage. The *ZO‐1* and *E‐Cadherin* levels significantly decreased in the OVA group in contrast with the NC group (Figure S5H,I).

We next assessed whether butyrate counteracts these effects in a concentration‐dependent manner. IEC‐6 cells were exposed to OVA to induce Notch activation and barrier damage, followed by treatment with 0.25–5 mM butyrate (Figure S6A). Butyrate at 0.25–1 mM reduced ROS levels and restored GSH levels as well as SOD and CAT activities, whereas 5 mM butyrate showed no antioxidant efficacy (Figure S6B–E). Importantly, 0.25–5 mM butyrate did not induce cytotoxicity (Figure S4F). Notably, OVA‐induced upregulation of *Jagged1* and *Hes1* mRNA was suppressed by 0.25–1 mM butyrate but paradoxically exacerbated by 5 mM butyrate (Figure S6F,G). The mRNA of TJs protein expression (*ZO‐1* and *E‐Cadherin*) was restored in the 0.5–1 mM butyrate groups but remained unaffected at higher concentrations (2–5 mM) (Figure S6H,I). These findings demonstrate that low‐dose butyrate (0.5–1 mM) ameliorates intestinal barrier integrity by suppressing OS‐driven Notch signaling, aligning with in vivo observations.

### Butyrate attenuated H₂O₂‐induced Notch activation via ROS suppression

To generalize the link between OS and Notch signaling, IEC‐6 cells were treated with 200 μM H₂O₂ (Figure S7A). Compared with the NC group, co‐treatment with 0.75 mM butyrate alleviated H₂O₂‐induced ROS accumulation and restored GSH levels along with SOD and CAT activities (Figure S7B–E). H₂O₂ treatment notably enhanced nuclear translocation of Notch intracellular domain (NICD) and upregulated protein and mRNA expression of *Jagged1* and *Hes1* (Figure S7F–L). In contrast, butyrate effectively inhibited H₂O₂‐induced Notch activation, as evidenced by reduced NICD nuclear localization and downregulated *Jagged1* and *Hes1* expression (Figure S7F–L). Notably, butyrate repaired the intestinal barrier impairment caused by H₂O₂ (Figure S7M,N). These results suggested that H_2_O_2_‐induced OS activates Notch signaling and disrupts the intestinal barrier, while butyrate reverses these effects.

### ROS and Notch pathway inhibitors confirm butyrate's mechanistic dependence on Notch signaling

**Figure 7:**
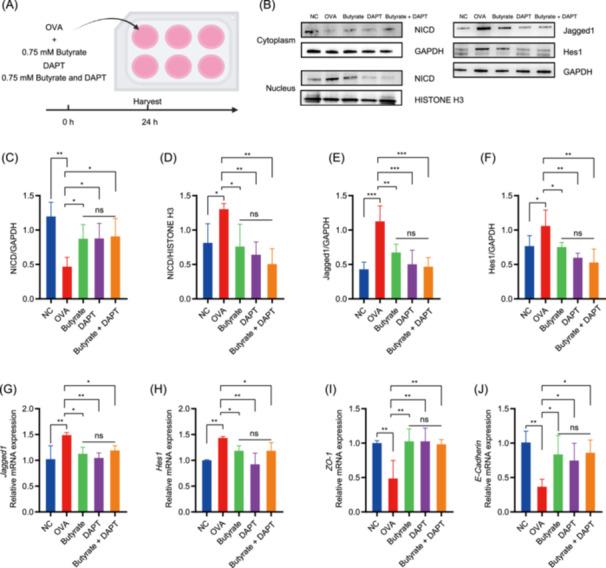
Effect of butyrate on the Notch signaling in IEC‐6 cells. (A) IEC‐6 cells were treated with butyrate (0.75 mM), N‐[N‐(3,5‐difluorophenacetyl)‐l‐alanyl]‐S‐phenylglycine t‐butyl ester (DAPT), or their combination ± 200 μg/mL OVA for 24 h. (B) Representative western blot images of Notch intracellular domain (NICD), GAPDH (cytosolic marker), HISTONE H3 (nuclear marker), Jagged1, and Hes1. The protein expressions of cytosolic NICD (C), nuclear NICD (D), total Jagged1 (E), and total Hes1 (F). The mRNA expressions of *Jagged1* (G), *Hes1* (H), *ZO‐1* (I), and *E‐Cadherin* (J). NC, negative control group; OVA, OVA group; Butyrate, 0.75 mM butyrate; DAPT, DAPT group; Butyrate + DAPT, butyrate and DAPT group, *n* = 3. *p < 0.05; **p < 0.01; ***p < 0.001.

Further validation using ROS and Notch inhibitors demonstrated the role of Notch signaling in butyrate's protective mechanism. IEC‐6 cells were treated with OVA in the presence or absence of Eukarion (EUK)‐134 or N‐[N‐(3,5‐difluorophenacetyl)‐ l‐alanyl]‐S‐phenylglycine t‐butyl ester (DAPT) (Figure S8A). 10 µM EUK‐134 or 40 μM DAPT did not induce cytotoxicity (Figure S4G). EUK‐134, a synthetic antioxidant, suppressed OVA‐induced ROS elevation and restored antioxidant defenses (Figure S8B–E), concurrently inhibiting Notch activation (*Jagged1*/*Hes1*, Figure S8F,G) and enhancing TJs protein (*ZO‐1*/*E‐Cadherin*, Figure S8H,I). In contrast, DAPT, a γ‐secretase inhibitor, directly inhibited Notch signaling (Figure S8F,G) and rescued TJs protein (Figure S8H,I) without modulating ROS or antioxidant systems (Figure S8B–E). IEC‐6 cells were treated with butyrate, DAPT, or their combination with OVA (Figure 7A). Notably, the combination of butyrate with DAPT did not further reverse NICD nuclear translocation suppression or enhance barrier integrity restoration beyond DAPT monotherapy (Figure 7B–J), demonstrating that butyrate's therapeutic effects are mediated through Notch signaling modulation. These findings showed that OVA‐induced barrier dysfunction is mediated by OS‐driven Notch overactivation, which is counteracted by low‐dose butyrate through modulation of this signaling pathway.

## DISCUSSION

SCFAs present within the intestinal contents may exert a significant influence on immune function, thereby playing a critical role in FA [16]. Previous studies have identified significant differences in SCFAs concentrations between allergic and the nonallergic individuals. For instance, the concentrations of acetate, propionate, and butyrate concentrations were substantially lower in the allergic group compared to the nonallergic group [17]. Moreover, children with the highest butyrate levels were also less likely to be diagnosed with FA [18]. The concentration of fecal pentanoate at 1 year of age was negatively correlated with FA at 13 years old [19]. Furthermore, infants with FA at 12 months of age had lower concentrations of acetic acid and succinic acid in their plasma at 4 months of age compared to healthy infants [20]. Currently, there is limited research that compares the levels of SCFAs in fecal samples among healthy individuals, FST, and FA individuals. Consistent with our observations, levels of propionic acid, acetic acid, valeric acid, and butyric acid were found to be reduced in individuals with FST and FA compared to HC. Notably, no significant disparities were observed between the FST and FA groups. Experimental data derived from an OVA‐sensitized murine model showed that butyrate had superior efficacy in relieving allergic symptoms and reducing OVA‐sIgE levels, outperforming acetate, propionate, and pentanoate. Moreover, in the effector phase of FA, mast cells and basophils play a crucial role as key effector cells.

FcεRI crosslinking mediated by food antigens and IgE activates effector cells, triggering degranulation with the consequent release of histamine, mMCP‐1, and other inflammatory cytokines that drive disease manifestations [21]. Butyrate also more effectively reduces serum levels of histamine and mMCP‐1. FA is often characterized by an overactive Th2 immune response. The loss of intestinal epithelial integrity enhances antigen uptake and promotes Th2‐type allergic responses by activating group 2 innate lymphoid cells (ILC2s), mast cells, basophils, and dendritic cells [22]. Butyrate inhibited the production of Th2 cytokines and increased the number of Foxp3^+^ Tregs, consistent with previous findings [23]. The results illustrated that butyrate upregulated the expression of TJs and *Muc2* gene in the jejunum. Additionally, butyrate could promote the expression of *Muc2* and TJs protein in IECs. DAO enzyme mainly exists in intestinal mucosal epithelial cells of the small intestine. Its serum concentration directly correlates with the integrity and maturity of IECs and is an important indicator of intestinal barrier function [24]. In this study, results demonstrated that the serum activity of DAO in the Butyrate group was significantly higher than that in the OVA group. d‐LA, a gut microbiota‐derived metabolite, enters systemic circulation when mucosal injury elevates intestinal permeability [25]. The absence of d‐LA elevation in OVA‐induced models indicates preserved integrity of d‐LA‐associated microbial consortia unaffected by ovalbumin exposure.

Changes in the gut microbiome are linked to the rising prevalence of FA. Microbiome dysbiosis leads to impaired gut barrier function, thereby increasing exposure to allergens and immune activation [26]. Here, we found alterations in the gut microbial structure of mice induced by OVA. OVA decreased the relative abundance of *L. murinus* while increasing that of *A. muciniphila*. Studies have proposed that *L. murinus* exerted protective impacts on intestinal permeability through restoration of the TJs protein, and decreased lipopolysaccharide (LPS) levels [27]. *A. muciniphila*, a symbiotic bacterium residing in the mucus layer, is primarily known for regulating host metabolic functions and immune responses to influence human health and disease [28]. However, *A. muciniphila* degrades intestinal mucus and proliferates on the mucosal surface, diminishing mucus layer thickness and exposing IECs directly to allergens [29]. Research demonstrated that combining *A. muciniphila* colonization with dietary fiber deficiency enhances innate type‐2 immunity and anti‐commensal IgE coating, exacerbating FA severity [30]. In this study, supplementation with butyrate reversed the gut microbiota structure and supported the maintenance of intestinal barrier integrity, thereby reducing allergens exposure and absorption, lessening immune activation, and mitigating allergic responses.

According to the UPLC‐MS results, we found that butyrate supplement enhanced anti‐inflammatory and antioxidant metabolites. For example, gentiopicroside, 6‐Gingerol, and OEA have been demonstrated to possess antioxidant, immunomodulatory properties, and anti‐inflammatory properties [31, 32, 33]. Study confirmed that ICA could reduce ROS levels and inflammatory factors expression in endothelial cells [34]. Moreover, OEA played a significant role in the regulation of host metabolism in the intestinal microbiota [35]. N‐acetylserotonin (NAS) has the functional features of neuroprotective, antioxidant, and antiapoptotic effects [36]. Tryptophan metabolites produced by intestinal flora are believed to contribute to the regulation of intestinal immune homeostasis [37]. In this study, OVA exposure reduced levels of tryptophan metabolites, including ICA, 5‐HIAA, N‐Me‐5HT, NAS, and KYNA; however, these metabolites were restored upon butyrate supplementation. Our preliminary study found that these metabolites are antiallergenic.

Dietary antigen tolerance initiation occurs through small intestinal absorption. Our study revealed that butyrate administration downregulated Notch signaling transcriptionally in IECs. This pathway activates when adjacent cell ligands engage Notch receptors, inducing structural modifications in the extracellular domain (NECD) that enable ADAM10‐mediated cleavage. Following γ‐secretase‐mediated cleavage, the released NICD migrates to the nucleus and assembles a transcriptional complex with CBF1‐Suppressor of Hairless‐LAG‐1 (CSL), Mastermind‐like (MAML), and associated cofactors to drive target gene activation [38]. This pathway is critically involved in sustaining intestinal barrier integrity [39]. Jagged1, a Notch ligand, regulates the expression of the Notch target gene *Hes1*, which subsequently influences goblet cell formation and MUC expression [10, 40, 41]. However, excessive activation of Notch signaling can damage the intestinal barrier, including TJs and the mucosal barrier, whereas Notch inhibitors can improve disuccinimidyl suberate‐induced colitis by restoring the intestinal barrier function [41, 42]. Previous research utilizing murine FA models investigated how Notch pathway inhibitors modulate antigen‐triggered hypersensitivity responses. Notch signaling contributes to the differentiation and accumulation of mucosal mast cells in the intestinal mucosa [10]. Another study demonstrated that suppressing Notch signaling alleviates antarctic krill tropomyosin (AKTM)‐induced FA by regulating gut microbiota and restoring Th1/Th2 balance [40]. In this study, we recognized that butyrate treatment decreased transcriptional levels of the Notch signaling pathway. Notch signaling pathway‐related proteins Hes1 and Jagged1 were significantly downregulated in mouse IECs treated with butyrate, accompanied by increased expression of *Muc2* and TJs‐associated genes in IECs. OVA induced mitochondrial dysfunction, exacerbating OS [43]. In the study, OVA increased ROS levels and decreased antioxidant molecule and enzymes, these changes were reversed following butyrate intervention. Moreover, it is known that high levels of exogenous ROS upregulate *Notch1* [44]. The data suggest that OVA‐induced ROS production led to mucosal barrier damage by activating the Notch signaling pathway.

Our study established a causal link between OS‐driven Notch signaling activation and intestinal barrier dysfunction. OVA exposure triggered ROS overproduction and Notch pathway activation (*Jagged1*/*Hes1* upregulation), concurrent with TJs protein (*ZO‐1* and *E‐cadherin*) suppression in IEC‐6 cells. EUK‐134, a synthetic mimetic of superoxide dismutase and catalase, reversed OVA‐induced ROS accumulation [45]. As expected, EUK134 attenuated Notch activation and restored TJs integrity. Prior evidence has demonstrated that antioxidants mitigate Notch signaling [46]. Importantly, DAPT served as a γ‐secretase inhibitor to suppress Notch signaling activation [10]. In the present study, DAPT directly suppressed the Notch pathway and repaired TJs protein without altering ROS and antioxidant molecules and enzymes, positioning the Notch signaling as a downstream effector of OS in barrier disruption. Butyrate exhibited a biphasic effect contingent on concentration. Low doses of butyrate (0.25–1 mM) attenuated ROS‐mediated Notch activation and rescued TJs protein. However, high doses of butyrate (2–5 mM) upregulated ROS levels and Notch signaling pathway. A previous study showed that butyrate at low concentrations (1 mM) protected cells from H_2_O_2_‐induced injury, while at high concentrations (8 and 16 mM), it caused inflammation and cell apoptosis [47]. This shows that high‐dose butyrate might induce OS in IECs, triggering activation of Notch signaling. Moreover, upon H₂O₂‐induced OS, 0.75 mM butyrate not only significantly alleviated oxidative damage but also suppressed Notch signaling activation, concomitantly enhancing intestinal epithelial TJs integrity. Importantly, the combination of butyrate with DAPT did not demonstrate stronger preservation of the intestinal barrier when compared to either treatment administered individually. As both butyrate and DAPT act through Notch pathway suppression, their combination may reach maximal inhibition thresholds. Our study demonstrates that low doses of butyrate ameliorate FA by inhibiting intestinal barrier damage mediated by the activation of Notch signaling under OS.

## CONCLUSION

Children with FST and FA exhibited lower fecal SCFAs levels, particularly butyric acid, compared to healthy children. Data from an OVA‐sensitized mouse model indicated that butyrate had a far more remarkable effect on alleviating allergic reactions than other SCFAs. This study illustrated that butyrate decreased the production of ROS and then inhibited the Notch signaling pathway in IECs, increasing gut barrier function. Collectively, our findings illustrate the potential mechanism of butyrate in modulating Notch, which may support butyrate‐producing dietary fibers, prebiotics, and probiotics for the prevention or treatment of FA.

## METHODS

### Study subjects and sample collection

A total of 60 individuals (0–18 years old) were recruited from the gastroenterology department at Children's Hospital of Nanjing Medical University (Nanjing, China) and stratified into three groups: children with (1) non‐atopic HC (*n* = 20), (2) FST (*n* = 20), and (3) FA (*n* = 20). Informed consent was obtained from parents with assent from children. This study received approval from the ethics committee of the Children's Hospital of Nanjing Medical University (approval 202405002‐1).

FA was characterized by a parental survey of a diagnosed allergy to egg, wheat, milk, soy, sesame, peanut, crab, shrimp, or nuts with an IgE ≥ 0.35 kU_A_/L to the same food. FST involved a food allergen‐sIgE level of ≥0.35 kU_A_/L, but without any history of adverse reactions to the same food [48]. HC had no history of food sensitization or food allergy at any time. Participants or their parents/guardians utilized sterile, screw‐cap fecal containers to collect fecal samples according to the instructions of research personnel. Fecal samples were collected, immediately frozen at −80°C, and stored until use.

### Mice

Three‐week‐old female mice (BALB/c, specific‐pathogen‐free (SPF)) were purchased from the Beijing Vital River Laboratory Animal Technology Co., Ltd. Mice were housed in a facility with a controlled light cycle (12 h light/12 h dark), temperature (25 ± 2°C), and humidity level (60 ± 5%). The Animal Care and Use Committee of Southeast University consented to the experimental protocol (Approval No.20230301040).

### Food allergy model

After the mice were acclimated to their environment for 1 week, they were randomly divided into six groups: NC group, OVA group, butyrate group, acetate group, propionate group, and pentanoate group (*n* = 8, per group). As previously described [49], mice were intraperitoneally injected with 50 μg OVA (A5503; Sigma‐Aldrich) plus 1 mg aluminum potassium sulfate as an adjuvant (A7210; Sigma‐Aldrich) on Days 0 and 14. 50 mg OVA was administered orally on Days 28, 30, 32, 34, and 36. Concentrations of 200 mM (sodium butyrate), 100 mM (acetate), 100 mM (propionate), and 100 mM (pentanoate) were administered in drinking water (Figure 1E).

### Anaphylaxis symptoms score

Anaphylaxis symptoms were assessed 1 h after the last OVA oral challenge based on criteria from previous studies, with the following scores: 0 = no symptoms; 1 = scratching and rubbing around the nose and head; 2 = reduced activity; 3 = wheezing, labored respiration and cyanosis around the mouth and the tail; 4 = no activity after prodding or tremor and convulsion; and 5 = death [50].

### Immunoglobulin assays

Blood samples were collected at the end of the protocol and centrifuged at 3000 rpm for 15 min. The levels of OVA‐sIgE (CAT#CSB‐E08914m, CUSABIO, China), histamine (CAT#E‐EL‐0032; Elabscience), and mMCP‐1 (CAT#88‐7503‐22; Invitrogen) were analyzed on serum samples by ELISA according to the manufacturer's protocol.

### Cytokine assays

The spleen was collected to obtain cell suspension. Then, cells were treated with 200 μg/mL OVA at 37°C for incubation [50]. After 72 h, supernatants were harvested and the concentrations of IL‐4 (CAT#CSB‐E04634m; CUSABIO), IL‐13 (CAT#CSB‐E04602m; CUSABIO), and IL‐10 (CAT#CSB‐E04594m; CUSABIO) were measured by using commercial ELISA Kits.

### Flow cytometry

Spleen cell suspensions were plated in 6‐well culture plates and subjected to 4 h stimulation with 50 ng/mL phorbol‐12‐myristate‐13‐acetate (PMA) (CAT#P8139; Sigma‐Aldrich) and 1 μg/mL of ionomycin (CAT#I3909; Sigma‐Aldrich), co‐administered with 1 mg/mL brefeldin A (CAT#B5936; Sigma‐Aldrich) [51]. Cellular suspensions underwent sequential labeling: first with FITC‐CD4 (CAT#553046; BD Biosciences) and PE‐CD25 (CAT#553075; BD Biosciences) in the dark conditions (30 min, 4°C), followed by intracellular staining using the Foxp3 Transcription Factor buffer set (CAT#00‐5523‐00; eBioscience). Final staining employed Alexa Fluor 647‐Foxp3 (CAT#560401; BD Biosciences), APC‐IFN‐γ (CAT#554413; BD Biosciences), and PE‐IL‐4 (CAT#554435; BD Biosciences). Fluorescence data analysis was performed in FlowJo v10.8.1.

### Evaluation of intestinal permeability

Immunofluorescence staining was performed to localize ZO‐1, Occludin, and Muc2 expression in jejunal mucosa. Mouse jejunal specimens were fixed in 4% paraformaldehyde under room temperature (RT) and paraffin‐embedded. Sectioned at 4 μm thickness for parallel antigen labeling: primary antibodies targeting ZO‐1, Occludin, and Muc2 were treated with overnight incubation (4°C), and incubated with secondary antibody exposure (50 min, RT) in the dark. Nuclear counterstaining employed 4',6‐diamidino‐2‐phenylindole (DAPI) before image acquisition using an inverted fluorescence microscope, with subsequent fluorescence quantification via Image‐J software.

The levels of DAO (CAT#A088‐3‐1; Nanjing Jiancheng) and d‐LA in serum were determined using commercial assay kits.

### H&E and AB‐PAS

Mouse jejunum specimens were fixed in 4% paraformaldehyde at RT and paraffin‐embedded. Sections (4 μm) were generated and subjected to histological staining protocols: H&E and AB‐PAS stains.

### Rat basophil leukemia cells (RBL‐2H3)

Serum specimens from four egg‐allergic patients (Wolcavi Biotech Co., Ltd.) exhibited egg‐sIgE concentrations of 26.5, 30.4, 32.1, and 25.7 KU_A_/L. RBL‐2H3 cells (ATCC) were cultured in MEM supplemented with 15% FBS under standard conditions (37°C, 5% CO₂). Cells were sensitized with 1:25 diluted patient serum (24 h exposure), followed by 1 h treatment with 50 μM Gentiopicroside, 6‐Gingerol, OEA, ICA, 5‐HIAA, N‐Me‐5HT, and NAS, then challenged with 30 µg/mL OVA challenge for 1 h [52]. β‐Hexosaminidase release was quantified [53]: in 96‐well plates, 60 μL supernatant combined with 60 μL p‐nitrophenyl‐N‐acetyl‐β‐d‐glucosaminide substrate (1 mM, pH 4.5) (37°C, 1.5 h). Reaction termination employed 150 μL Na₂CO₃‐NaHCO₃ buffer (0.1 M, pH 10). Absorbance (405 nm) determined release percentage relative to controls: β‐hexosaminidase release (%) = 100% × (absorbance sample‐absorbance media)/(absorbance normal‐absorbance media).

### Isolation of IECs

Mice were euthanized, and small intestines were collected for IECs isolation. As previously described [5, 54], tissues were immersed in cold RPMI 1640 + 3% FBS. Then, after the excision of fat tissues and Peyer's patches, intestines were longitudinally incised, segmented into 1 cm pieces, and rinsed twice with cold PBS. Tissue fragments were incubated in RPMI 1640 + 3% FBS containing 1 mM EDTA (CAT#C0196; Beyotime) and 1 mM DTT (CAT#ST043; Beyotime), being shaken (200 rpm, 37°C, 25 min). Cell suspensions were filtered (100 µm cell strainer), then centrifuged (1600 rpm, 6 min, 4°C), and precipitation (IECs) was collected.

### Quantitative real‐time PCR (qRT‐PCR)

Total RNA was extracted from IECs, IEC‐6, and RBL‐2H3 cells using the Total RNA Kit (Cat# R711‐01; Vazyme) according to the manufacturer's protocol. 1 μg of RNA was reverse‐transcribed with the HiScript® Strand cDNA Synthesis Kit (Cat# R323‐01; Vazyme). Quantitative PCR (qPCR) was performed using the ChamQ Universal SYBR qPCR Master Mix (Cat# Q712‐02; Vazyme). Relative expressions of target genes were analyzed using the 2−ΔΔCt method with β‐actin as the endogenous housekeeping gene. Primers obtained from Sangon Biotech are listed in Table S2.

### Rat small intestine crypt epithelial cells (IEC‐6)

IEC‐6 cells (ATCC) were maintained in Dulbecco's Modified Eagle Medium containing 10% fetal bovine serum (FBS) at 37°C and 5% CO_2_. IEC‐6 cells were cultured in 6‐well plates and exposed to 200 μg/mL OVA for 24 h, with or without 10 μM EUK‐134 (CAT# HY‐100594; MCE) or 40 μM DAPT (MCE). IEC‐6 cells were incubated for 24 h with different concentrations of butyrate (0.25, 0.5, 0.75, 1, 2, and 5 mM) with or without 200 μg/mL OVA. Subsequently, the cells were collected for protein and RNA extraction, ROS, antioxidant molecule, and enzymes determination.

### Induction of OS in IEC‐6 cells by H₂O₂

IEC‐6 cells were then incubated with butyrate (0.75 mM) for 1 h as a pretreatment. Then, H_2_O_2_ (200 μM) was added and the cells were cultured for 4 h [55]. Subsequently, the cells were collected for protein and RNA extraction, and for the determination of ROS, antioxidant molecules, and enzymes.

### Antioxidant assays

The OS parameters of the small intestine tissues, serum, and cells lysate, including SOD (CAT#BC516; Solarbio), GSH (CAT#BC1175; Solarbio), and CAT (CAT#BC4785; Solarbio) levels, were determined using commercial detection kits according to the instructions. For ROS levels, IECs, RBL‐2H3, and IEC‐6 cells were collected and incubated with 10 μM DCFH‐DA (Cat# S0034S; Beyotime) for 20 min and then analyzed by flow cytometry.

### Statistical analysis

Continuous variables are expressed as mean ± SD (normally distributed) or medians with interquartile ranges (IQR) (non‐normal); categorical variables as frequencies (%). For continuous variables of between‐group comparisons were performed using the Student's *t*‐test for two groups or one‐way analysis of variance (ANOVA) with the least significant difference post hoc test for more than two groups. The chi‐square test was utilized for categorical variables. Body weight measurements across multiple time points were analyzed using repeated‐measures ANOVA. *p* less than 0.05 was considered significant. Data were analyzed by SPSS 25 software (SPSS Inc.) for statistical tests.

## AUTHOR CONTRIBUTIONS

**Jialu Shi**: Conceptualization; methodology; software; writing—original draft; investigation; formal analysis; writing—review and editing; validation; data curation; supervision. **Wenjun Mao**: Writing—review and editing; conceptualization; methodology; software. **Yuqing Song**: Validation. **Yuxin Wang**: Validation. **Lili Zhang**: Validation. **Yan Xu**: Investigation; formal analysis. **Huiwen Gu**: Investigation; validation. **Siyu Yao**: Writing—review and editing; methodology. **Yuanhang Yao**: Writing—review and editing; methodology. **Zhifeng Liu**: Resources; supervision. **Vijaya Raghavan**: Writing—review and editing; resources. **Jin Wang**: Conceptualization; methodology; project administration; resources; supervision; writing—review and editing; funding acquisition.

## CONFLICT OF INTEREST STATEMENT

The authors declare no conflicts of interest.

## ETHICS STATEMENT

This study received approval from the ethics committee of the Children's Hospital of Nanjing Medical University (No. 202405002‐1) and the Animal Care and Use Committee of Southeast University (No. 20230301040). Informed consent was obtained from all participants before their involvement in the study.

## Supporting information


**Figure S1.** Short‐chain fatty acids (SCFAs) concentrations of children's fecal samples were evaluated.
**Figure S2.** Butyrate exhibited immunoregulatory effects in OVA‐sensitized mice.
**Figure S3.** Fecal samples of mice metabolomic analysis by ultra‐performance liquid chromatography‐mass spectrometry (UPLC‐MS).
**Figure S4.** The viability levels of RBL‐2H3 and IEC‐6 cells were determined by counting kit‐8 assay (CCK8) assay.
**Figure S5.** OVA‐induced oxidative stress to activate Notch signaling.
**Figure S6.** Effect of different concentrations of butyrate on the Notch signaling in rat small intestine crypt epithelial cells (IEC‐6) cells.
**Figure S7.** Effect of butyrate on the Notch signaling in IEC‐6 cells under oxidative stress.
**Figure S8.** Inhibition of oxidative stress could inhibit Notch signaling.

**Table S1.** Characteristics of study population.
**Table S2.** Primer sequences used in RT‐qPCR assays.

## Data Availability

The data that support the findings of this study are available on request from the corresponding author. The data are not publicly available due to privacy or ethical restrictions. All the sequencing data have been deposited in GSA under BioProject accession number PRJCA037068 (16S rRNA) (https://ngdc.cncb.ac.cn/bioproject/browse/PRJCA037068) and PRJCA036993 (RNA sequencing) (https://ngdc.cncb.ac.cn/bioproject/browse/PRJCA036993). The Metabolomics data reported in this paper have been deposited in the OMIX, China National Center for Bioinformation/Beijing Institute of Genomics, Chinese Academy of Sciences (accession no. OMIX009398, https://ngdc.cncb.ac.cn/omix/select-edit/OMIX009398). The data and scripts used are saved in GitHub (https://github.com/Sjl012/Butyrate_FA_iMeta/tree/master). Supplementary materials (figures, methods, tables, graphical abstract, slides, videos, Chinese translated version, and update materials) may be found in the online DOI or iMeta Science http://www.imeta.science/.
