# Supplementary material for: Butyrate alleviates food allergy by improving intestinal barrier integrity through suppressing oxidative stress‐mediated Notch signaling
Source: Imeta. 2025 Apr 3;4(3):e70024. doi: 10.1002/imt2.70024 (PMC12130580; doi:10.1002/imt2.70024)
Supplement: Supplementary file 1 — Figure S1. Short‐chain fatty acids (SCFAs) concentrations of children's fecal samples were evaluated. Figure S2. Butyrate exhibited immunoregulatory effects in OVA‐sensitized mice. Figure S3. Fecal samples of mice metabolomic analysis by ultra‐performance liquid chromatography‐mass spectrometry (UPLC‐MS). Figure S4. The viability levels of RBL‐2H3 and IEC‐6 cells were determined by counting kit‐8 assay (CCK8) assay. Figure S5. OVA‐induced oxidative stress to activate Notch signaling. Figure S6. Effect of different concentrations of butyrate on the Notch signaling in rat small intestine crypt epithelial cells (IEC‐6) cells. Figure S7. Effect of butyrate on the Notch signaling in IEC‐6 cells under oxidative stress. Figure S8. Inhibition of oxidative stress could inhibit Notch signaling. [file IMT2-4-e70024-s001.docx]

**Supporting information to**

**Butyrate alleviates food allergy by improving intestinal barrier integrity through suppressing oxidative stress-mediated Notch signaling**

**Running title**: Butyrate alleviated food allergy through inhibition Notch signaling

Jialu Shi^1#^, Wenjun Mao^2#^, Yuqing Song^1^, Yuxin Wang^1^, Lili Zhang^1^, Yan Xu^1^, Huiwen Gu^1^, Siyu Yao^1^, Yuanhang Yao^1^, Zhifeng Liu^3^, Vijaya Raghavan^4^, Jin Wang^1^*

^1^Key Laboratory of Environmental Medicine and Engineering, Ministry of Education, Department of Nutrition and Food Hygiene, School of Public Health, Southeast University, Nanjing 210009, China

^2^Department of Thoracic Surgery, The Affiliated Wuxi People's Hospital of Nanjing Medical University, Wuxi People's Hospital, Wuxi Medical Center, Naniing Medical University, Wuxi 214023, China

^3^Department of gastroenterology, Children's Hospital of Nanjing Medical University, Nanjing 210029, China

^4^Department of Bioresource Engineering, Faculty of Agricultural and Environmental Sciences, McGill University, Sainte-Anne-de-Bellevue, QC H9X3V9, Canada

^#^These authors contributed equally: Jialu Shi, Wenjun Mao

*Correspondence: [jin.wang6@mail.mcgill.ca](mailto:jin.wang6@mail.mcgill.ca) (Jin Wang)

**Methods**

## Determination of short-chain fatty acids (SCFAs) concentration in children's fecal samples

Freeze-dried fecal samples were weighed and mixed with 500 µL of saturated NaCl (containing 0.5% phosphoric acid). The mixture was vortexed for 10 min, and 1 mL ether was added, followed by centrifugation at 12,000 rpm and 4 °C for 15 min. The supernatant was filtered through a 0.22 μm filter and transferred to a glass vial. The concentration of SCFAs was normalized against the initial stool weight. SCFAs analysis was performed on a GC-MS/MS (TQ8040 NX, Shimadzu Corporation, Japan) equipped with an Agilent DB-FFAP (30m × 0.25mm × 0.25um). The GC conditions were as follows: initial temperature of 90 °C (1 min)-120 °C (2 min) at 5 °C/min, 120 °C (2 min)-150 °C (0 min) at 10 °C/min, 150 °C (0 min)-200 °C (0.5 min) at 20 °C/min; injection port temperature, 230 °C; carrier gas, helium (99.999% purity); flow rate, 1 mL/min; split ratio, 10:1; injection volume, 1 μL. The MS operating conditions were as follows: ionization source temperature, 280°C; interface temperature, 230 °C. A triple-quadrupole equipped with an EI source and operated in MRM mode was used for detection after separation on an Agilent DB-FFAP column.

## 16S rRNA gene sequencing for fecal samples of mice

Mouse fecal DNA was isolated via the TGuide S96 Magnetic Soil/Stool DNA Kit according to the manufacturer’s protocols. The full-length 16S rRNA gene was amplified with primer pairs 27F: AGRGTTTGATYN TGGCTCAG and 1492R: TASGGHTACCTTGTTASGACTT. The amplicons were quantified, after which the normalized equimolar concentrations of amplicons were pooled and sequenced on the PacBio Sequel II platform. The raw reads generated from sequencing were filtered and demultiplexed using the SMRT Link software (version 8.0) to obtain the circular consensus sequencing (CCS) reads. Sequences with similarity > 97% of CCS were clustered into the same operational taxonomic unit (OTU) by USEARCH (v10.0). The Alpha diversity was calculated and displayed by the QIIME2 and R software, respectively. Principal coordinate analysis (PCoA) was used to analyze the beta diversity. Beta diversity was determined using QIIME to evaluate the degree of similarity of microbial communities from different samples. Furthermore, we employed Linear Discriminant Analysis (LDA) effect size (LEfSe) to test the significant taxonomic difference among groups.

## Ultra-performance liquid chromatography-mass spectrometry (UPLC-MS)

The mouse feces samples were accurately added into Methanol-Acetonitrile -water (2:2:1), vortexed, ground, and sonicated. Waters Xevo G2-XS QTOF high-resolution mass spectrometer can collect primary and secondary mass spectrometry data in MSe mode under the control of the acquisition software (MassLynx V4.2, Waters). In each data acquisition cycle, dual-channel data acquisition can be performed on both low collision energy and high collision energy at the same time. The low collision energy is 2 V, the high collision energy range is 10-40V, and the scanning frequency is 0.2 s for a mass spectrum. The parameters of the ESI ion source are as follows: capillary voltage: 2000 V (positive ion mode) or 1500 V (negative ion mode); cone voltage: 30 V; ion source temperature: 150 °C; desolvent gas temperature 500 ℃; backflush gas flow rate: 50 L/ h; Desolventizing gas flow rate: 800 L/h. After normalizing the original peak area information with the total peak area, the follow-up analysis was performed. Principal component analysis and Spearman correlation analysis were used to judge the repeatability of the samples within the group and the quantity control samples. The identified compounds are searched for classification and pathway information in Kyoto Encyclopedia of Genes and Genomes (KEGG), human metabolome database (HMDB), and lipid maps databases. According to the grouping information, calculate and compare the difference multiples, T-test was used to calculate the difference significance *p*-value of each compound. The R language package ropls was used to perform orthogonal partial least squares-discriminant analysis (OPLS-DA) modeling, and 200 times permutation tests were performed to verify the reliability of the model. The VIP value of the model was calculated using multiple cross-validation. The method of combining the difference multiple, the *p* value, and the variable projection importance (VIP) value of the OPLS-DA model was adopted to screen the differential metabolites. The screening criteria are fold change (FC) > 1, *p* value ≤ 0.05, and VIP > 1. The difference metabolites of KEGG pathway enrichment significance were calculated using a hypergeometric distribution test.

## Transcriptional profiling by RNA sequencing

RNA was isolated from small intestinal intestinal epithelial cells (IECs) using the TRIZOL method. RNA concentration and purity were measured using NanoDrop 2000 (Thermo Fisher Scientific, Wilmington, DE). RNA integrity was assessed using the RNA Nano 6000 Assay Kit of the Agilent Bioanalyzer 2100 system (Agilent Technologies, CA, USA). Sequencing libraries were generated using Hieff NGS Ultima Dual-mode mRNA Library Prep Kit for Illumina (Yeasen Biotechnology (Shanghai) Co., Ltd.) following manufacturer’s recommendations and index codes were added to attribute sequences to each sample. All samples were sequenced using an Illumina NovaSeq. Gene expression levels of the transcripts were computed by fragments per kilobase of transcript per million mapped reads (FPKM). Differential expression analysis of two samples was determined using the DESeq2_EBSeq. The false discovery rate (FDR) < 0.05 and FC ≥ 1.5 were considered as significantly differentially expressed. Gene expression differences were visualized using a volcano plot. The x-axis represents log_2_ (FC) for gene expression, and the y-axis represents -log_10_ (FDR). KOBAS database and cluster Profiler software were used to test the statistical enrichment of differential expression genes in KEGG pathways.

## Western Blot Analysis

To separate nuclear and cytoplasmic fractions, cells were processed using the Nuclear and Cytoplasmic Protein Extraction Kit (Cat# P0028, Beyotime, China). Total protein was extracted using RIPA buffer (Cat# P0013B, Beyotime, China) supplemented with 1 mM phenylmethanesulfonyl fluoride (PMSF) (Cat# ST506, Beyotime, China), 1 × protease inhibitor cocktail, and 1 × phosphatase inhibitor cocktail (Cat# P1045, Beyotime, China) for 30 min on ice. Protein concentrations were determined by bicinchoninic acid (BCA) assay (Cat# P0010, Beyotime, China). Equal amounts of proteins were resolved on 10% sodium dodecyl sulfate polyacrylamide gel electrophoresis (SDS-PAGE) gels and transferred to polyvinylidene fluoride (PVDF) membranes (Merck Millipore, Germany). After blocking with 5% skim milk powder in tris-buffered saline with tween 20 (TBST) for 1 h at RT, membranes were incubated with primary antibodies at 4 °C overnight: anti-NICD (1:1000, Cat# 4147, Cell Signaling Technology, USA), anti-Jagged1 (1:1000, Cat# JF96-06, HUABIO, China), and anti-Hes1 (1:1000, Cat# A0925, ABclonal, China) were applied for 1 h at room temperature‌ (RT). Signals were developed using enhanced chemiluminescence (ECL) reagent (Cat# BL520A, Biosharp, China). GAPDH (1:10000, Cat# ab181602, Abcam, UK) antibodies were used as cytoplasm and total protein loading control, and HISTONE H3 (1:2000, Cat# 17168-1-AP, Proteintech, USA) was used as nuclear loading control for cellular fractionation immunoblotting analysis. The western blotting data were quantified using Image J software.


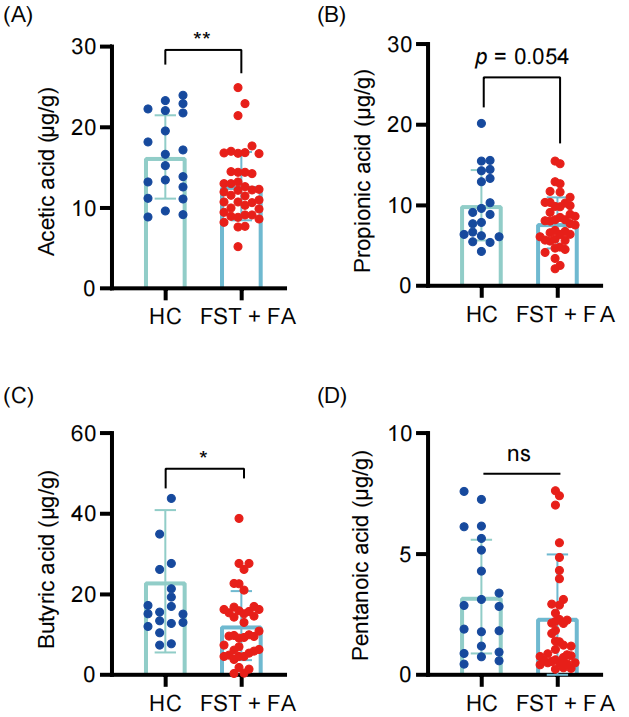


**Figure S1 Short-chain fatty acids (SCFAs) concentrations of children's fecal samples were evaluated.** (A-D) The concentrations of acetic acid (A), propionic acid (B), butyric acid(C), and pentanoic acid (D) in HC and FST + FA groups. HC, healthy controls group, *n* = 20; FST + FA, food-sensitized tolerance + food allergy group, *n* = 40. **p* < 0.05; ***p* < 0.01; ****p* < 0.001.


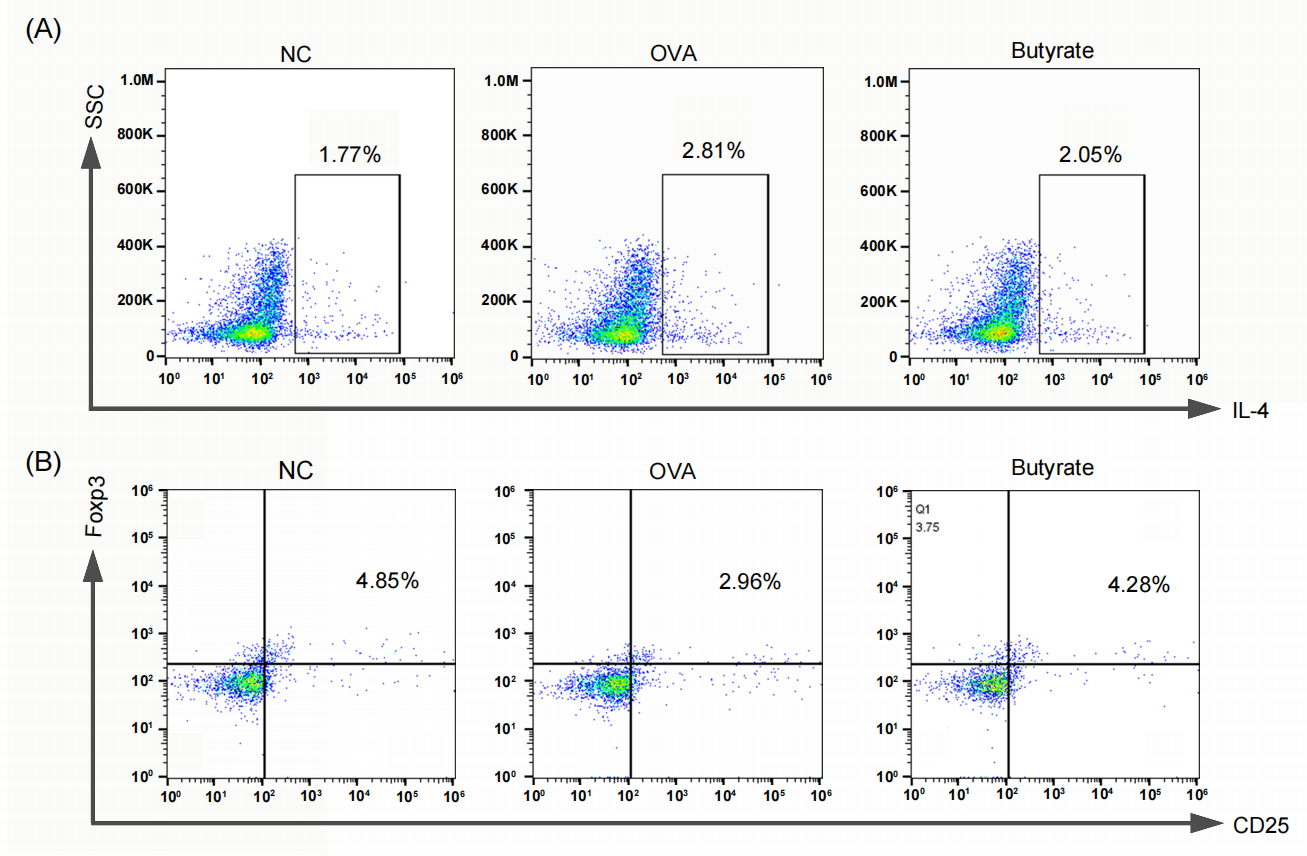


**Figure S2 Butyrate exhibited immunoregulatory effects in ovalbumin (OVA) mice.** After the last challenge, the spleen was collected. (A-B) Interleukin (IL)-4-producing CD4^+^ T cells (A) and CD4^+^CD25^+^Foxp3^+^ Treg cells (B) numbers of splenocytes were measured by flow cytometry after stimulation with phorbol-12-myristate-13-acetate (PMA)/ionomycin in the presence of brefeldin A, *n* = 3. NC, negative control group; OVA, OVA group; Butyrate, butyrate group; Acetate, acetate group; Propionate, propionate group; Pentanoate, pentanoate group.

**Figure S3 Fecal samples of mice metabolomic analysis by ultra-performance liquid chromatography-mass spectrometry (UPLC-MS).** (A) Principal component analysis (PCA) showed that all fecal metabolites were significantly distinct in all groups. (B) Scores and permutation test of Orthogonal Projections to Latent Structures-Discriminant Analysis (OPLS-DA). (C-D) Significant differentially abundant metabolites between two groups: NC vs OVA (C) and OVA vs Butyrate (D) (*p* value < 0.05, fold changes (FC) >1 and variable projection importance (VIP) > 1) in the volcano plot. NC, negative control group; OVA, OVA group; Butyrate, butyrate group, *n* =5.

**Figure S4 The viability levels of RBL-2H3 and IEC-6 cells were determined by counting kit-8 assay (CCK8).** (A-C) RBL-2H3 cells were treated with various concentrations of key metabolites (0-300 µM) (A), OVA (0-50 µg/mL) (B), and egg-allergic patient serum (1:15-1:25) (C) for 24 h, followed by incubation with CCK8 solution for 20 min. (D) RBL-2H3 cells were incubated with egg-allergic patient serum at varying dilution ratios (1:15 to 1:25) for 24 h, subsequently exposed to OVA at concentrations of 30 or 40 µg/mL for 1 h, and then treated with CCK-8 solution for 20 min. (E) The release of β-hexosaminidase in the cell supernatant was subsequently quantified to determine the optimal molding concentration. (F) IEC-6 cells were treated with different concentrations of butyrate (0.25-5 mM) for 24 h, followed by incubation with CCK8 solution for 20 min. (G) IEC-6 cells were treated 200 µg/mL with or without 10 µM Eukarion-134 (EUK-134) or 40 μM N-[N-(3,5-difluorophenacetyl)-L-alanyl] -S-phenylglycine t-butyl ester (DAPT) for 24 h, followed by incubation with CCK8 solution for 20 min. NC, negative control group; OVA, OVA group, *n* = 3. **p* < 0.05; ** *p* < 0.01; *** *p* < 0.001.


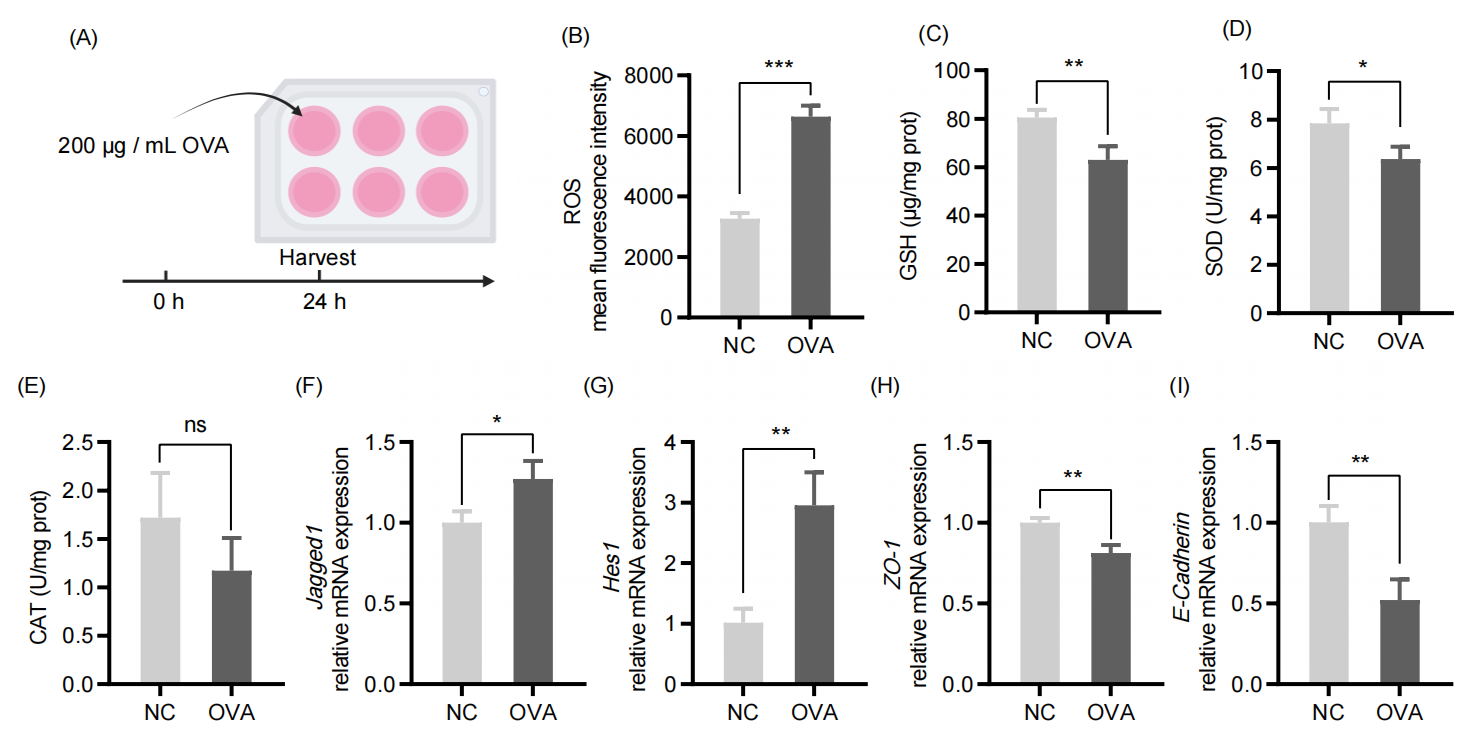


**Figure S5 OVA-induced oxidative stress to activate Notch signaling.** (A) IEC-6 cells were seeded into 6-well plates and incubated for 24 h with 200 μg/mL OVA. (B) Reactive oxygen species (ROS) mean fluorescence intensity was measured by Flow Cytometry. The expressions of anti-oxidant-related proteins were quantified. (C-E) The levels of glutathione (GSH) (C), superoxide dismutase (SOD) (D), and catalase (CAT) (E). (F-G) The mRNA expressions of Notch signaling proteins *Jagged1* (F) and *Hes1* (G). (H-I) The mRNA expressions *ZO-1* (H) and *E-Cadherin* (I) in IECs. NC, negative control group; OVA, OVA group, *n* = 3. **p* < 0.05; ** *p* < 0.01; *** *p* < 0.001.

**
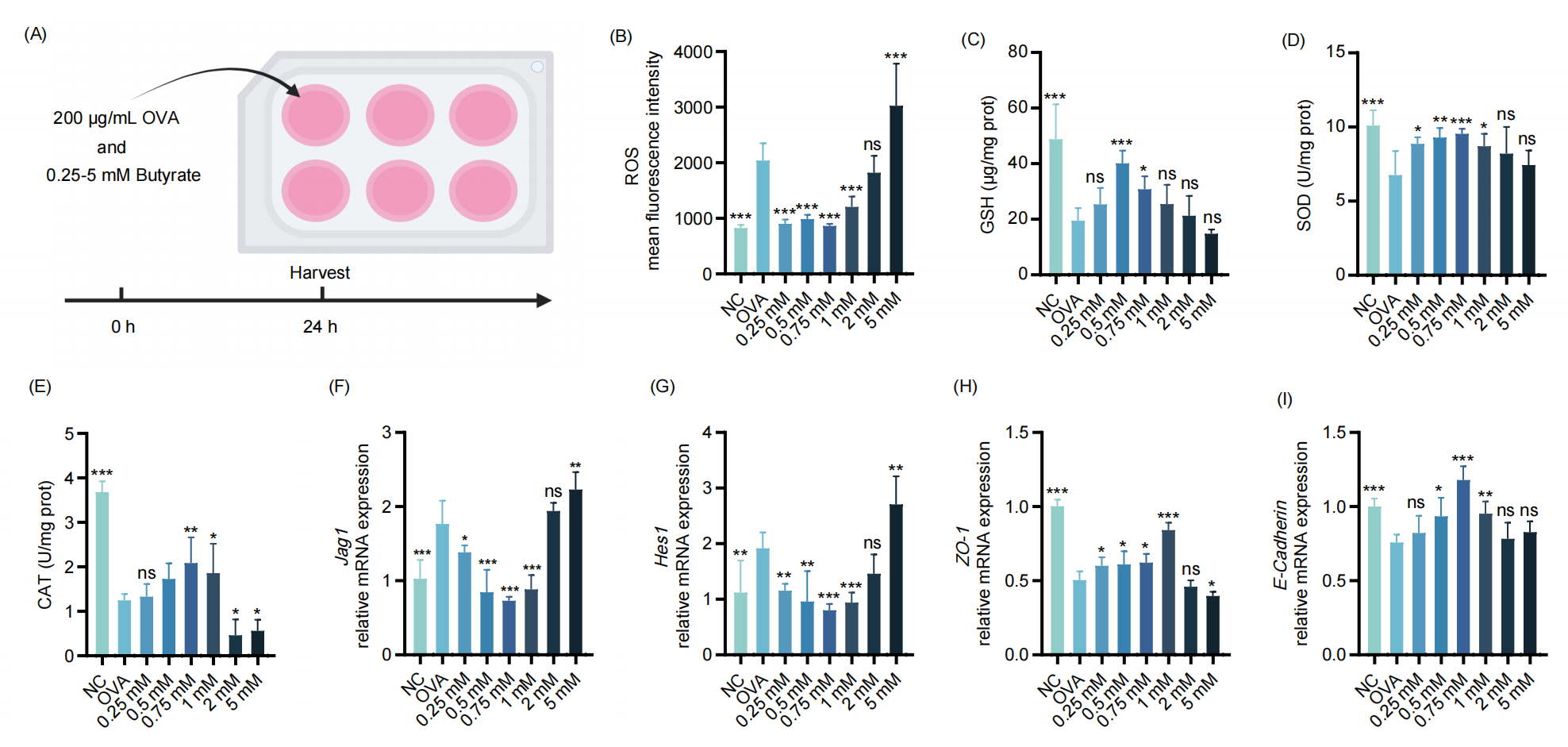
**

**Figure S6 Effect of different concentrations of butyrate on the Notch signaling in rat small intestine crypt epithelial cells (IEC-6) cells.** (A) IEC-6 cells were seeded into 6-well plates and incubated for 24 h with different concentrations of butyrate (0.25, 0.5, 0.75, 1, 2, and 5 mM) with or without 200 μg/mL OVA. (B) ROS mean fluorescence intensity was measured by Flow Cytometry. The expressions of anti-oxidant-related proteins were quantified. (C-E) The levels of GSH (C), SOD (D), and CAT (E) in IEC-6 cells lysate. (F-G) The mRNA expressions of Notch signaling proteins *Jagged1* (F) and *Hes1* (G). (H-I) The mRNA expressions *ZO-1* (H) and *E-Cadherin* (I) in IECs. NC, negative control group; OVA, OVA group; 0.25 mM, 0.25 mM butyrate; 0.5 mM, 0.5 mM butyrate; 0.75 mM, 0.75 mM butyrate; 1 mM, 1 mM butyrate; 2 mM, 2 mM butyrate; 5 mM, 5 mM butyrate, *n* = 4. **p* < 0.05; ** *p* < 0.01; *** *p* < 0.001.

**Figure S7 Effect of butyrate on the Notch signaling in IEC-6 cells under oxidative stress.** (A) IEC-6 cells were then pretreated with butyrate (0.75 mM) for 1 h. Then, H_2_O_2_ (100 μM) was added and the cells were cultured for 4 h. (B) ROS mean fluorescence intensity was measured by Flow Cytometry. The expressions of anti-oxidant-related proteins were quantified. (C-E) The levels of GSH (C), SOD (D), and CAT (E) in IEC-6 cells lysate. (F) Representative Western blot images of Notch intracellular domain (NICD), GAPDH (cytosolic marker), HISTONE H3 (nuclear marker), Jagged1, and Hes1. (G) Cytosolic NICD levels. (H) Nuclear NICD levels. (I) Total Jagged1 protein expression. (J) Total Hes1 protein expression. (K-N) The mRNA expressions *Jagged1* (K), *Hes1* (L), *ZO-1* (M), and *E-Cadherin* (N) in IECs. NC, negative control group; H_2_O_2_, H_2_O_2_ group; Butyrate, 0.75 mM butyrate group, *n* = 3. **p* < 0.05; ** *p* < 0.01; *** *p* < 0.001.

**
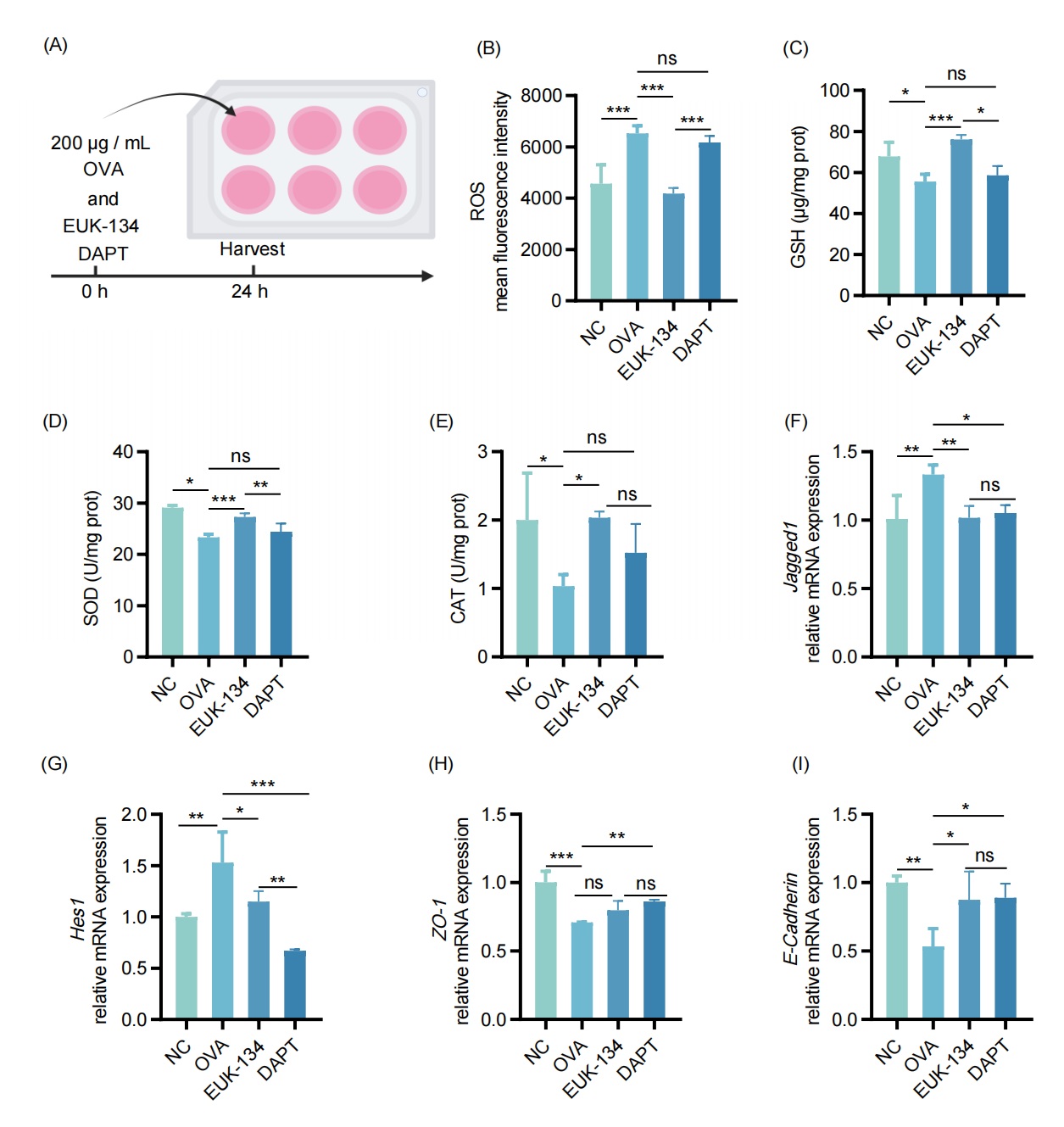
**

**Figure S8 Inhibition of oxidative stress could inhibit Notch signaling.** (A) IEC-6 cells were seeded into 6-well plates and incubated for 24 h with 200 μg/mL OVA in the presence or absence of 10 μM Eukarion (EUK)-134 or 40 μM N-[N-(3,5-difluorophenacetyl)- L-alanyl]-S-phenylglycine t-butyl ester (DAPT). (B) ROS mean fluorescence intensity was measured by Flow Cytometry. (C-E) The expressions of anti-oxidant-related proteins were quantified. The levels of GSH (C), SOD (D), and CAT (E). (F-G) The mRNA expressions of Notch signaling proteins *Jagged1* (F) and *Hes1*(G). (H-I) The mRNA expressions *ZO-1* (H) and *E-Cadherin* (I) in IECs. NC, negative control group; OVA, OVA group. EUK-134, EUK-134 group; DAPT, DAPT group, *n* = 3. **p* < 0.05; ** *p* < 0.01; *** *p* < 0.001.
